# An Eco-Friendly Improved Protocol for the Synthesis of Bis(3-indolyl)methanes Using Poly(4-vinylpyridinium)hydrogen Sulfate as Efficient, Heterogeneous, and Recyclable Solid Acid Catalyst

**DOI:** 10.1155/2013/616932

**Published:** 2013-09-02

**Authors:** Janardhan Banothu, Rajitha Gali, Ravibabu Velpula, Rajitha Bavantula, Peter A. Crooks

**Affiliations:** ^1^Department of Chemistry, National Institute of Technology, Warangal, Andhra Pradesh 506 004, India; ^2^Department of Pharmaceutical Sciences, College of Pharmacy, University of Arkansas for Medical Sciences, Little Rock, AR 72205, USA

## Abstract

Highly efficient and eco-friendly protocol for the synthesis of bis(3-indolyl)methanes by the electrophilic substitution reaction of indole with aldehydes catalyzed by poly(4-vinylpyridinium)hydrogen sulfate was described. Excellent yields, shorter reaction times, simple work-up procedure, avoiding hazardous organic solvents, and reusability of the catalyst are the most obvious advantages of this method.

## 1. Introduction

Indole derivatives have emerged as important class of nitrogen containing heterocycles and are known to possess broad spectrum of biological and pharmacological activities [1, 2]. In particular, bis(indolyl)methanes (BIMs) which are isolated from terrestrial and marine natural sources such as parasitic bacteria, tunicates, and sponge are found as possible antibacterial, anticarcinogenic, genotoxic, and DNA-damaging agents [3]. BIMs are active cruciferous substances for promoting estrogen metabolism [4] and have the ability to prevent cancer by modulating certain cancer-causing estrogen metabolites [5].

Owing to their diverse biological properties, many methods have been developed for their synthesis using various catalytic systems such as amberlyst-15 [6], iodine [7], boric acid [8], fluoroboric acid [9], sulfamic acid [10], NbCl_5_ [11], silica sulfuric acid [12], cellulose sulfuric acid [13], zeolite [14], ceric ammonium nitrate [15], polyvinylsulfonic acid [16], dodecylsulfonic acid [17], dodecylbenzenesulfonic acid [18], HClO_4_-SiO_2_ [19], ZrOCl_2_·8H_2_O [20], Dy(OTf)_3_ [21], protic solvent [22], and ionic liquids [23]. However, most of these reported methods suffer from one or several drawbacks such as low yields, prolonged reaction times, use of hazardous, expensive, moisture-sensitive, and large quantity of reagents, involving harsh reaction conditions, tedious workup procedure, and difficulty in recovery, and reusability of the catalysts. Therefore, still there is a need to develop an efficient, eco-friendly, and versatile method for the synthesis of bis(indolyl)methanes.

In continuation of our research towards the synthesis of biologically important molecules using novel methodologies [24], we report herein a simple, highly efficient, and eco-friendly method for the synthesis of bis(3-indolyl)methanes using poly(4-vinylpyridinium)hydrogen sulfate [P(4-VPH)HSO_4_] [25] as heterogeneous and reusable solid acid catalyst. 

## 2. Results and Discussion

**Scheme 1:**
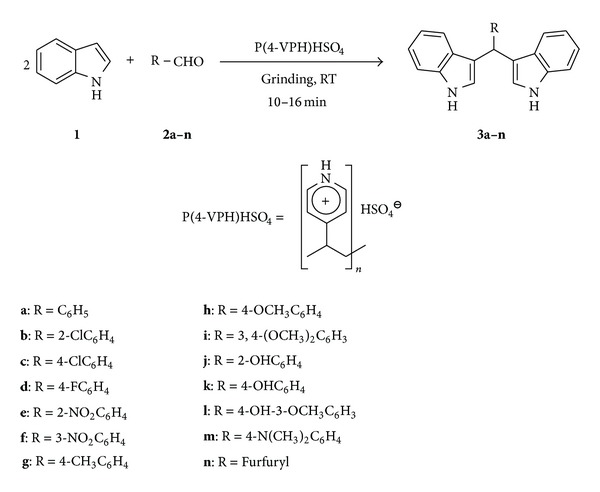
P(4-VPH)HSO_4_ catalyzed synthesis of bis(3-indolyl)methanes.

The electrophilic substitution reaction of indole with aryl aldehydes catalyzed by P(4-VPH)HSO_4_ is shown in Scheme 1. The reaction smoothly proceeds at room temperature under grinding technique to provide the corresponding bis(3-indolyl)methane with good yields in shorter reaction times.

**Scheme 2:**
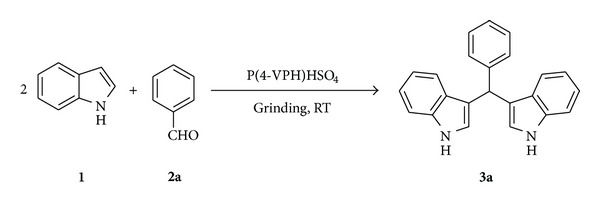
Synthesis of bis(3-indolyl)phenylmethane catalyzed by P(4-VPH)HSO_4_.

In order to synthesize bis(3-indolyl)methanes under solvent-free conditions, a model reaction was performed between indole and benzaldehyde using P(4-VPH)HSO_4_ as catalyst (Scheme 2). Indole (2 mmol) and benzaldehyde (1 mmol) were taken in a mortar and ground at room temperature with pestle by the different amount of catalyst (Table 1). After completion of the reaction shown by TLC (monitored every 2 min), we observed 94% yield in 12 min in the presence of 15 mg of the catalyst. Decreasing the amount of the catalyst results in; low yield of the product (**3a**) even after prolonged reaction times than the higher amount of catalyst does not show any effect on product yield and reaction time. At the optimized conditions (15 mg of catalyst, grinding at room temperature), the reaction was carried out with substituted aldehydes and the corresponding bis(3-indolyl)methanes were obtained in good yields (Table 2). All the synthesized compounds were well characterized by their analytical and spectral studies and compared with the literature values.

We investigated the efficiency of P(4-VPH)HSO_4_ compared to other acid catalysts based on the synthesis of bis(indol-3-yl)phenylmethane (**3a**). The results show that P(4-VPH)HSO_4_ is an efficient catalyst in terms of product yield and reaction time (Table 3). The catalyst was recovered after completion of the reaction; the catalyst was washed with dichloromethane, dried, and reused for subsequent reactions for additional five times. We observed a slight decrease in its activity in terms of product yield (Table 4).

**Scheme 3:**
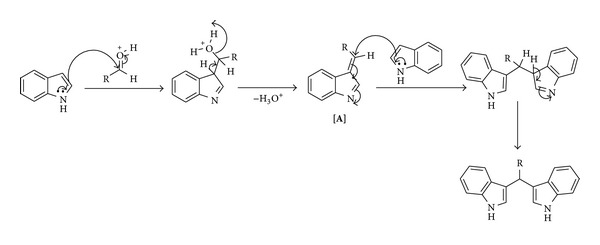
Plausible mechanism for the formation of bis(3-indolyl)methanes catalyzed by P(4-VPH)HSO_4_.

A plausible mechanism for the formation of bis(3-indolyl)methanes catalysed by P(4-VPH)HSO_4_ is proposed in Scheme 3. In the presence of catalyst, the electrophilicity of carbonyl carbon has increased and it readily reacts with indole, affording the corresponding 3-arylidine-3*H*-indole [**A**] *via* dehydration. Intermediate [**A**] on reaction with second mole of indole followed by rearrangement affords the final product in good yield. 

## 3. Experimental

All the reagents and solvents were purchased from Aldrich/Merck and used without further purification. Melting points were determined in open capillaries using Stuart SMP30 apparatus and are uncorrected. The progress of the reactions as well as purity of compounds was monitored by thin layer chromatography with F_254_ silica-gel precoated sheets using hexane, ethyl acetate (8 : 2) as eluent; UV light and iodine vapors were used for detection. Products were characterized by comparison with authentic samples and by spectroscopy data (IR and ^1^H NMR). IR spectra were recorded on Perkin-Elmer 100S spectrometer using KBr disk, and values are expressed in cm^−1^. ^1^H NMR spectra were recorded with Bruker 400 MHz spectrometer and chemical shifts are expressed in ppm. Elemental analyses were performed on a Carlo Erba modal EA1108 and Mass spectra were recorded on a Jeol JMSD-300 spectrometer. 

### 3.1. General Procedure for the Synthesis of Bis(3-indolyl)methanes (**3a–n**)

Poly(4-vinylpyridinium)hydrogen sulfate (15 mg) was added to a mixture of indole (2 mmol) and aryl aldehyde (1 mmol) in a mortar and ground with a pestle in appropriate time as shown in Table 2. After completion of the reaction monitored by TLC, 5 mL of water was added and stirred at room temperature for additional 5 min. Thus, the solid obtained was filtered, washed with water, dried, and recrystallized from ethanol to afford the analytically pure product. Aqueous layer containing catalyst was recovered under reduced pressure, washed with dichloromethane, dried, and reused for subsequent reactions.

### 3.2. Spectral Data for Selected Compounds

#### 3.2.1. Bis(3-indolyl)-4-chlorophenylmethane (**3c**)

IR (KBr) *υ*_max⁡_ (cm^−1^): 3472 (NH), 1598 (C=C), 678 (C–Cl); ^1^H NMR (400 MHz, DMSO-*d*_6_): *δ* 5.87 (s, 1H), 6.62 (s, 2H), 7.02–7.72 (m, 12H), 7.91 (s, 2H); MS (ESI) *m/z*: 357 (M+H)^+^; Anal. Calcd. for C_23_H_17_ClN_2_: C, 77.41; H, 4.80; N, 7.85; Found: C, 77.62; H, 4.57; N, 7.93.

#### 3.2.2. Bis(3-indolyl)-3,4-dimethoxyphenylmethane (**3i**)

IR (KBr) *υ*_max⁡_ (cm^−1^): 3478 (NH), 1604 (C=C), 1035 (C–O–C); ^1^H NMR (400 MHz, DMSO-*d*_6_): *δ* 3.76 (s, 3H), 3.85 (s, 3H), 5.85 (s, 1H), 6.43–6.52 (m, 1H), 6.52 (d, *J* = 8.0 Hz, 1H), 6.70 (s, 2H), 6.78 (d, *J* = 8.0 Hz, 1H), 7.03–7.45 (m, 8H), 7.92 (s, 2H); MS (ESI) *m/z*: 405 (M + Na)^+^; Anal. Calcd. for C_25_H_22_N_2_O_2_: C, 78.51; H, 5.80; N, 7.32; Found: C, 78.70; H, 5.64; N, 7.51.

#### 3.2.3. Bis(3-indolyl)furylmethane (**3n**)

IR (KBr) *υ*_max⁡_ (cm^−1^): 3477 (NH), 1602 (C=C), 1093 (C–O–C); ^1^H NMR (400 MHz, DMSO-*d*_6_): *δ* 5.94 (s, 1H), 6.07 (d, *J* = 8.4 Hz, 1H), 6.72 (s, 2H), 7.03–7.48 (m, 10H), 7.96 (s, 2H); MS (ESI) *m/z*: 313 (M + H)^+^; Anal. Calcd. for C_21_H_16_N_2_O: C, 80.75; H, 5.16; N, 8.97; Found: C, 80.90; H, 5.33; N, 8.79.

## 4. Conclusion

In conclusion, we have developed a simple and efficient method for the preparation of bis(3-indolyl)methanes utilizing poly(4-vinylpyridinium)hydrogen sulfate as solid acid catalyst under solvent-free conditions at ambient temperature. This protocol offers several advantages in terms of product yield, operational simplicity, and reusability of the catalyst and it obeys the green chemistry conditions by avoiding hazardous organic solvents. We believe that this method is superior to existing methods. 

## Figures and Tables

**Table 1:** Optimizing the reaction conditions^a^.

Entry^a^	P(4-VPH)HSO_4_ (mg)	Time (min)	Yield^b^ (%)
1	—	60	Trace
2	5	30	51
3	10	30	76
4	15	12	94
5	20	12	94

^a^Reaction conditions: indole (2 mmol) and benzaldehyde (1 mmol), grinding at RT.

^b^Isolated yields of the product **3a**.

**Table 2:** Synthesis of bis(3-indolyl)methanes catalyzed by P(4-VPH)HSO_4_ under solvent-free conditions.

Entry^a^	Aldehyde	Product	Time (min)	Yield^b^ (%)	Melting points (°C)	
					Found	Lit. [Reference]
1	Benzaldehyde	**3a**	12	94	150–152	150–152 [7]
2	2-Chlorobenzaldehyde	**3b**	14	92	71–73	72–74 [7]
3	4-Chlorobenzaldehyde	**3c**	12	96	76–78	76-77 [7]
4	4-Fluorobenzaldehyde	**3d**	10	90	78–80	80–82 [9]
5	2-Nitrobenzaldehyde	**3e**	14	88	140–142	140-141 [18]
6	3-Nitrobenzaldehyde	**3f**	14	89	262–264	264-265 [20]
7	4-Methylbenzaldehyde	**3g**	10	92	95–97	94–96 [7]
8	4-Methoxybenzaldehyde	**3h**	10	92	186–188	187–189 [7]
9	3,4-Dimethoxybenzaldehyde	**3i**	10	94	195–197	197-198 [19]
10	2-Hydroxybenzaldehyde	**3j**	12	89	342–344	340–342 [18]
11	4-Hydroxybenzaldehyde	**3k**	10	90	124–126	124–126 [20]
12	4-Hydroxy-3-methoxybenzaldehyde	**3l**	10	91	126–128	126-127 [22]
13	4-(Dimethylamino)benzaldehyde	**3m**	14	90	168–171	170–172 [20]
14	Furan-2-carbaldehyde	**3n**	16	89	321–323	322–325 [7]

^a^Reaction conditions: indole (2 mmol), aldehyde (1 mmol), and P(4-VPH)HSO_4_ (15 mg), grinding at RT.

^b^Isolated yields.

**Table 3:** Comparing the efficiency of P(4-VPH)HSO_4_ with some reported acid catalysts for the synthesis of bis(3-indolyl)phenylmethane **(3a)**.

Entry^a^	Catalyst (g)	Reaction conditions	Time (min)	Yield (%) [reference]
1	Boric acid (0.0124 g)	Neat, 80°C	60	94 [8]
2	Oxalic acid (0.09 g)	H_2_O, 80°C	40	96 [26]
3	Sulfamic acid (0.0485 g)	MeOH, RT	180	90 [10]
4	PEG-SO_3_H (0.045 g)	H_2_O, RT	20	92 [27]
5	Dodecyl sulfonic acid (0.025 g)	H_2_O, RT	25	95 [17]
6	Polyvinylsulfonic acid (0.0216 g)	EtOH, RT	120	93 [16]
7	Silica sulfuric acid (0.1 g)	Neat, RT	40	92 [12]
8	Cellulose sulfuric acid (0.1 g)	Grinding, RT	8	93 [13]
9	P(4-VPH)HSO_4_ (0.015 g)	Grinding, RT	12	94 [present work]

Note: For comparison, mole percentages were converted into grams.

**Table 4:** Reusability of P(4-VPH)HSO_4_ for the synthesis of bis(3-indolyl)phenylmethane^a^.

Run	Cycle	Yield^b^ (%)
1	0	94
2	1	93
3	2	92
4	3	92
5	4	90
6	5	88

^a^Reaction conditions: indole (2 mmol), benzaldehyde (1 mmol), and P(4-VPH)HSO_4_ (15 mg), grinding at RT for 12 min.

^b^Isolated yields.
